# Supplementary material for: Cis-regulatory evolution of Wnt family genes contributes to a morphological difference between silkworm species
Source: PLoS Biol. 2026 Mar 10;24(3):e3003605. doi: 10.1371/journal.pbio.3003605 (PMC12974830; doi:10.1371/journal.pbio.3003605)
Supplement: Attachment — Submitted filename: Review EBR PLOS Bio Cis reg silworms.docx [file pbio.3003605.s024.docx]

**Review of the manuscript:** Cis-regulatory evolution of *Wnt*-family genes contributes

to a morphological difference between silkworm species

**Overall thoughts and background:**

In this manuscript, the authors aim to elucidate the genetic basis of caudal horn length in Bombycoidea moths. The authors describe how such work can provide insights into the genetic control/evolution of complex morphological traits, phenotypic divergence between closely related species, adaptation, and even reproductive isolation. More specifically, the authors use interspecific crosses between *Bombyx mori* and its closest relative*, B. mandarina, which exhibit different caudal horn lengths, to map the genetic differences underlying these* length differences. The authors were able to first identify eight QTL from which the three largest explain around one third of the mean horn length differences between species. The largest of these QTLs houses a Wnt-family cluster, which points the authors to the hypothesis that one or several of these *Wnt* genes might be controlling the differences in horn length. The authors were able to identify *Wnt1* and *Wnt6* as candidates due to their differential expression between the caudal horn and abdominal segment 8 in hybrids (*B. mandarina* allele expressed higher). This was validated across development via qPCR where *Wnt1 and Wnt6* are shown to be expressed higher in *B. mandarina* within the abdominal segment 8 and mostly across the time leading to molting. The authors functionally validated the role of *Wn1* and *Wnt6* in caudal horn length via CRISPR/Cas9-mediated knockouts. They were able to observe that knockouts of the *B. mandarina* alleles for both Wnt genes reduced horn length (*Wnt1* knockout having a stronger effect), showing their involvement in proper horn length.

In general, this manuscript is well put together, organized, and cohesive. The authors do a great job in guiding the reader and also following the logical steps associated with the specific questions. It provides very detailed and, to a certain degree, mechanistic information on the genetic control of the formation of complex structures (like the caudal horn) in a non-model system, which is very impressive. In summary, the authors showcase the data well, making clear both the findings and relevance. However, I want to point out a handful of major concerns with the presentation and analyses of some of the key datapoints. I outline some of those major concerns below with details on some minor concerns as well. I believe that a revised version of the manuscript tackling some of the general requests should merit publication.

**Major Concerns or Adjustments:**

1. **The first major concern is related to the Wnt1 and Wnt6 CRISPR/Cas9-mediated B. mandarina-allele-specific knockouts-** I want to highlight that this is an incredible experiment done by the authors and that can't be underscored. However, to my knowledge, the authors did not provide or show the images from which the quantifications were done. Given that the caudal horn is a morphologically complex structure, this quantification needs images highlighting the resulting phenotype or at least representative data used for the quantification. This is key for the unbiased analyses and to satisfy the curiosity of any reader of the manuscript. If images were recorded to perform the quantification, this should be an easy request to incorporate and will heavily enrich the overall impact of the experiments and manuscript. Such images could even be nested within the figure where the quantifications are done, in case the authors want to avoid making a new image.
2. **Did the authors ever notice an effect on other morphological metrics when knocking out Wn1 and Wnt6?** Again, as the author mentioned, this is a fleshy/complex structure, and I know the starting data details for the mapping are accounting variation based on length, but given their effect on that metric, I was wondering if the authors looked at any other metrics. Like measuring the circularity of the base of the caudal horn, or if there are any pigmentation changes (given the Wnt family's known history of co-regulating shape and pigmentation). Adding details about this would be informative, especially from the angle of learning about the genetic underpinnings of building complex morphological structures, which would line up with what the authors refer to in the introduction. This is also a relevant point for the discussion.

The authors point out a handful of times how we know a lot from Wnt genes controlling morphological divergence between species, mostly from examples in the pigmentation world. There are several scenarios in which such details also interconnect with appendage growth. I feel it is a missed opportunity that the authors did not report on pigmentation (at least for the CRISPR experiment), given that there are small pigmentation differences between *Bombyx mori* and *B. mandarina* based at least on the pictures shown in Figure 1*.* I also understand this request might come off as seeking out new analyses. I want to emphasize that showing images, as mentioned above, would already satisfy this by just adding a couple more lines into the results/discussion and providing the representative data to enhance the relevance of the findings more widely. Again, if not, other differences are noted, stating that those other metrics were lacking differences would be very informative.

**Minor Concerns or Adjustments:**

1. Line- 126 is one of the first places to include the word backcross at least for one of the first BC1 or a bit more details to ease with readability for this section.
2. Similarly to the major concern 1. I consider that more transparency on the way this structure (caudal horn) is being measured for the main text would be of benefit. Currently, Figure S1 is the only place where we see those relevant details, which are the starting base for all the work that follows. I would include that image or a version of it earlier in the main text before the difference is mapped, or bring it up in the same text location, but not as a supplemental file.
3. The text of the genotypes in Figure 4 is way too small. It was quite difficult to read them, even when zooming in.
4. Statement of line 235 is very interesting and relevant. Was this from data not shown by the authors, or is it lacking a reference?
5. Line 278: The statement can be more specific, highlighting time as well, given that the authors have more details about when the dynamics are changing.
6. Just want to bring to the author's attention that in *Heliconius* butterflies, it has recently been suggested that Wnt signaling can modulate both appendage extension and pigmentation via regulation of the gene *aristaless* (Bayala et al. 2023). I think this is a reference point that enhances several of the mechanistic claims presented by the authors and ties really well, given what we know about aristaless from *Bombyx* within its involvement in antennal branching (Ando et al, 2018).
7. Line 283- Would be good to break the statement between flies and butterflies.
8. Increased Wnt activity is associated with proliferation and cell growth in most documented scenarios, which may be a point to include in the discussion.
9. Line 351- Other species within Moths, or more widely?
10. Line 391- Details of the imaging setup? Are they present somewhere else?
11. Figures 2 and 4 would benefit from a bigger font.
