# Supplementary material for: Cis-regulatory evolution of Wnt family genes contributes to a morphological difference between silkworm species
Source: PLoS Biol. 2026 Mar 10;24(3):e3003605. doi: 10.1371/journal.pbio.3003605 (PMC12974830; doi:10.1371/journal.pbio.3003605)
Supplement: Attachment — Submitted filename: ResponsetoReviewers_12212025_AP_final.docx [file pbio.3003605.s026.docx]

Response to Reviewers for “Cis-regulatory evolution of *Wnt* family genes contributes to a morphological difference between silkworm species”

Kenta Tomihara^1,¶^, Ana Pinharanda^2,3,4,¶^, Young Mi Kwon^2^, Andrew M. Taverner^5^, Laura Kors^6^, Matthew L. Aardema^7^, Julia C. Holder^2^, Lin Poyraz^2^, Takashi Kiuchi^1,&^, and Peter Andolfatto^2,&^

¶ Contributed equally to this work

& Co-supervised this work

Corresponding authors. Email: [pa2543@columbia.edu](mailto:pa2543@columbia.edu), [kiuchi@g.ecc.u-tokyo.ac.jp](mailto:kiuchi@g.ecc.u-tokyo.ac.jp)

We thank the editor and reviewers for their constructive comments on our manuscript. We have revised the paper to address all comments and suggestions. A major point was the need to more clearly link quantitative measurements of horn length to overall horn morphology. To address this, we have better integrated into the main text the description of how caudal horn length was measured using landmarks, as illustrated in Fig. S1, which includes images of representative individuals from the parental species. In addition, we modified Fig. S7 to show representative individuals corresponding to *B. mandarina* chromosome 4 introgression lines (i.e., Chr4 “consomics”) and *B. mandarina*-allele-specific knock-outs. We also improved the logical flow between the main text and the Supplementary Information, increased font sizes for readability, and added details on imaging conditions.

In response to requests for greater biological context, we strengthened the Results and Discussion by adding temporal specificity to the cis-regulatory effects of *Wnt1* and *Wnt6*, clarifying the developmental window during which regulatory divergence is observed. Where reviewers asked about additional phenotypic effects (e.g. pigmentation or other morphological metrics), we now explicitly discuss the absence of detectable differences. We also ensured that all cis-regulatory claims are commensurate with the data, as requested by the Academic Editor.

Finally, we addressed all editorial and technical comments, including data deposition and accession numbers, DOI-linked data availability statements in figure legends, reference list review, figure permissions, and formatting requirements.

**EDITOR’S COMMENTS**

1. **Ensure cis-regulatory claims are commensurate with the data**

In the revised manuscript, we have clarified the scope of our conclusions to reflect that our data are consistent with a cis-regulatory mechanism. Specifically, we now mention that, at the L5 stage, the magnitude of allele-specific expression closely matches the total species-level expression differences (Lines 318-326).

This close correspondence indicates that cis-regulatory divergence is sufficient to explain most, if not all, of the observed species-level Wnt1 and Wnt6 expression differences. We have clarified this point in the text and explicitly labeled Fig. 3B as L5.

**REVIEWER #1 COMMENTS**

1. **Could benefit from pictures to help the reader connect the metrics to**

S1A Fig is intended to connect the quantitative metric we use with the overall horn morphology. While we previous referred to this figure in the Methods, we now refer to it in the Results (Lines 149-150), which should make things clearer. We now also show representative individuals corresponding to *B. mandarina* chromosome 4 introgression lines (i.e., Chr4 “consomics”) and *B. mandarina*-allele-specific knock-outs in Fig. S7. The raw images of all individuals for this experiment are also uploaded to the online database: <https://doi.org/10.5281/zenodo.17947265>. We believe these changes should help in better connecting our caudal horn measurements with observed changes in overall horn morphology.

**REVIEWER #2 COMMENTS**

**Major Concerns or Adjustments:**

1. **The first major concern is related to the Wnt1 and Wnt6 CRISPR/Cas9-mediated B. mandarina-allele-specific knockouts- I want to highlight that this is an incredible experiment done by the authors and that can't be underscored. However, to my knowledge, the authors did not provide or show the images from which the quantifications were done. Given that the caudal horn is a morphologically complex structure, this quantification needs images highlighting the resulting phenotype or at least representative data used for the quantification. This is key for the unbiased analyses and to satisfy the curiosity of any reader of the manuscript. If images were recorded to perform the quantification, this should be an easy request to incorporate and will heavily enrich the overall impact of the experiments and manuscript. Such images could even be nested within the figure where the quantifications are done, in case the authors want to avoid making a new image.**

We believe this comment is addressed in our response to Reviewer 1’s comments above.

2. **Did the authors ever notice an effect on other morphological metrics when knocking out Wn1 and Wnt6? Again, as the author mentioned, this is a fleshy/complex structure, and I know the starting data details for the mapping are accounting variation based on length, but given their effect on that metric, I was wondering if the authors looked at any other metrics. Like measuring the circularity of the base of the caudal horn, or if there are any pigmentation changes (given the Wnt family's known history of co-regulating shape and pigmentation). Adding details about this would be informative, especially from the angle of learning about the genetic underpinnings of building complex morphological structures, which would line up with what the authors refer to in the introduction. This is also a relevant point for the discussion.**

We address this comment jointly with the 3^rd^ below.

3. **The authors point out a handful of times how we know a lot from Wnt genes controlling morphological divergence between species, mostly from examples in the pigmentation world. There are several scenarios in which such details also interconnect with appendage growth. I feel it is a missed opportunity that the authors did not report on pigmentation (at least for the CRISPR experiment), given that there are small pigmentation differences between Bombyx mori and B. mandarina based at least on the pictures shown in Figure 1. I also understand this request might come off as seeking out new analyses. I want to emphasize that showing images, as mentioned above, would already satisfy this by just adding a couple more lines into the results/discussion and providing the representative data to enhance the relevance of the findings more widely. Again, if not, other differences are noted, stating that those other metrics were lacking differences would be very informative.**

We agree pleiotropic effects on morphology and pigmentation would be interesting to note.

In the CRISPR/Cas9-mediated allele-specific knockout experiments targeting *Wnt1* and *Wnt6*, we did not observe any effects on larval traits beyond caudal horn length. In particular, we did not detect qualitative changes in horn base shape, overall horn morphology, or larval pigmentation in knockout larvae compared to controls. We now explicitly state this lack of additional phenotypic effects in the Results (lines 143-145) and discuss its implications in the Discussion (lines 1386-1389). Also see new panel D on Figure S7.

As we noted on lines 464-471, previous experiments have linked Wnt1 expression to changes in larval morphology (Edayoshi et al 2015) and pigmentation (Yamaguchi et al 2013). However, those experiments involved ectopic expression of Wnt1, whereas our experiments involve knock-outs of the *B. mandarina* allele, which may resemble a global down-regulation of Wnt1 (see Fig 4). This difference may reconcile our findings with previous studies.

**Minor Concerns or Adjustments:**

1. **Line- 126 is one of the first places to include the word backcross at least for one of the first BC1 or a bit more details to ease with readability for this section.**

The detailed information of BC1 and F2 crosses are added in Lines 162-167.

2. **Similarly to the major concern 1. I consider that more transparency on the way this structure (caudal horn) is being measured for the main text would be of benefit. Currently, Figure S1 is the only place where we see those relevant details, which are the starting base for all the work that follows. I would include that image or a version of it earlier in the main text before the difference is mapped, or bring it up in the same text location, but not as a supplemental file.**

We have revised the main text to more clearly describe the measurement approach at the point where horn length differences are first introduced (lines 147-149, also see major comment replies above).

While we retain Figure S1 as a supplemental figure, we now explicitly reference it earlier in the Results (lines 149-152). In addition, we have updated the legend of Figure 2 to more clearly guide the reader to this morphological context. Together, we think these changes ensure that the basis for caudal horn quantification is transparent and accessible.

3. **The text of the genotypes in Figure 4 is way too small. It was quite difficult to read them, even when zooming in.**

We have increased the text font from 7 to 11

4. **Statement of line 235 is very interesting and relevant. Was this from data not shown by the authors, or is it lacking a reference?**

This is data was not shown, but we now include it and the methods used. Amino acid changes between *B. mori* and *B. mandarina* for each gene in the QTL region are now shown in S4 Table. In the Methods section, we described the approach used to evaluate whether genes within the QTL interval exhibit amino acid-level differences between two species (Lines 694-707).

5. **Line 278: The statement can be more specific, highlighting time as well, given that the authors have more details about when the dynamics are changing.**

We agree with the reviewer. Figure 3 indicates that this downregulation of *Wnt1* and *Wnt6* is restricted to the caudal horn tissue and occurs just before molting. However, the difference in horn length between *B. mori* and *B. mandarina* was already present in neonate larvae (S1B Fig), suggesting that this downregulation also occurred in the embryonic stage. We now mentioned this in Lines 359-361.

6. **Just want to bring to the author's attention that in Heliconius butterflies, it has recently been suggested that Wnt signaling can modulate both appendage extension and pigmentation via regulation of the gene aristaless (Bayala et al. 2023). I think this is a reference point that enhances several of the mechanistic claims presented by the authors and ties really well, given what we know about aristaless from Bombyx within its involvement in antennal branching (Ando et al, 2018).**

Thank you very much for this valuable information. We referenced these papers in the revised manuscript (Lines 366-372).

7. **Line 283- Would be good to break the statement between flies and butterflies.**

We clarified this in lines 363-366.

8. **Increased Wnt activity is associated with proliferation and cell growth in most documented scenarios, which may be a point to include in the discussion.**

Thank you for the suggestion. In the revised manuscript, we explicitly added that *Wnt* signaling is broadly associated with cell proliferation and tissue growth (Lines 366-371).

9. **Line 351- Other species within Moths, or more widely?**

We clarified our statement. Since the caudal horn is only present in some Lepidopteran species, comparative studies focusing on this trait will be limited to a restricted group of species. However, in addition to caudal horns, insects have an impressive range of protruding structures, such as setae, spines, helmet, and head horns. Thus, we consider that comparative studies should be performed in both within and beyond Lepidoptera (Lines 497-499).

10. **Line 391-** **Details of the imaging setup? Are they present somewhere else?**

Details of the imaging setup are documented in Lines 543-544.

11. **Figures 2 and 4 would benefit from a bigger font.**

We agree and have increased the text font from 7 to 11

**REVIEWER #3 COMMENTS**

1. **Without a more in-depth molecular analysis of actual cis-regulatory elements mediating Wnt levels, it is not particularly enlightening in terms of biological mechanism. It is quite well accepted that regulatory differences can be responsible for phenotypic divergence—the authors even include a Table with other examples. While there continues to be debate over the extent to which regulatory vs. coding differences have the largest impact, the basic role of regulatory changes is not disputed. There are even other examples of Wnt involvement. Therefore, while these data may be exciting to those particularly interested in lepidopteran morphology or developmental roles of Wnt genes, I don't find this study to be of high interest generally.**

We thank Reviewer #3 for reviewing our manuscript. We agree that cis-regulatory evolution of developmental regulators, including Wnt genes, has been documented across multiple systems, and we do not argue that cis-regulatory evolution of Wnt genes is unprecedented.

However, the conclusion drawn by the reviewer rests on grouping together mechanistically and phenotypically distinct classes of traits. As made explicit in Table S7 (previously S6) and now clarified in the revised Discussion (lines 365-391 and 405-408), previously published examples of Wnt cis-regulatory evolution involve pigmentation patterning traits. By contrast, cases in which cis-regulatory changes at Wnt ligands are directly linked to divergence in a three-dimensional morphological structure between species has not previously been reported.

Our study therefore does not claim novelty on the basis of gene identity alone, but on the demonstrated role of tissue-specific cis-regulatory changes at Wnt1 and Wnt6 in shaping a complex morphological structure. Table S7 was included to provide transparent context for this claim, not to imply equivalence among fundamentally different trait categories.
